# Neutralizing Human Antibodies against Severe Acute Respiratory Syndrome Coronavirus 2 Isolated from a Human Synthetic Fab Phage Display Library

**DOI:** 10.3390/ijms22041913

**Published:** 2021-02-15

**Authors:** Yu Jung Kim, Min Ho Lee, Se-Ra Lee, Hyo-Young Chung, Kwangmin Kim, Tae Gyu Lee, Dae Young Kim

**Affiliations:** New Drug Development Center, Osong Medical Innovation Foundation, Cheongju-si, Chungcheongbuk-do 28160, Korea; yjkim@kbiohealth.kr (Y.J.K.); hpimh3@kbiohealth.kr (M.H.L.); srlee@kbiohealth.kr (S.-R.L.); hchung@kbiohealth.kr (H.-Y.C.); kwangmin.kim@kbiohealth.kr (K.K.); tglee17@gmail.com (T.G.L.)

**Keywords:** SARS-CoV-2, spike protein, receptor-binding domain, phage display, monoclonal antibody

## Abstract

Since it was first reported in Wuhan, China, in 2019, the severe acute respiratory syndrome coronavirus 2 (SARS-CoV-2) has caused a pandemic outbreak resulting in a tremendous global threat due to its unprecedented rapid spread and an absence of a prophylactic vaccine or therapeutic drugs treating the virus. The receptor-binding domain (RBD) of the SARS-CoV-2 spike protein is a key player in the viral entry into cells through its interaction with the angiotensin-converting enzyme 2 (ACE2) receptor protein, and the RBD has therefore been crucial as a drug target. In this study, we used phage display to develop human monoclonal antibodies (mAbs) that neutralize SARS-CoV-2. A human synthetic Fab phage display library was panned against the RBD of the SARS-CoV-2 spike protein (SARS-2 RBD), yielding ten unique Fabs with moderate apparent affinities (*EC*_50_ = 19–663 nM) for the SARS-2 RBD. All of the Fabs showed no cross-reactivity to the MERS-CoV spike protein, while three Fabs cross-reacted with the SARS-CoV spike protein. Five Fabs showed neutralizing activities in in vitro assays based on the Fabs’ activities antagonizing the interaction between the SARS-2 RBD and ACE2. Reformatting the five Fabs into immunoglobulin Gs (IgGs) greatly increased their apparent affinities (*K*_D_ = 0.08–1.0 nM), presumably due to the effects of avidity, without compromising their non-aggregating properties and thermal stability. Furthermore, two of the mAbs (D12 and C2) significantly showed neutralizing activities on pseudo-typed and authentic SARS-CoV-2. Given their desirable properties and neutralizing activities, we anticipate that these human anti-SARS-CoV-2 mAbs would be suitable reagents to be further developed as antibody therapeutics to treat COVID-19, as well as for diagnostics and research tools.

## 1. Introduction

Since the pandemic outbreak of the severe acute respiratory syndrome coronavirus 2 (SARS-CoV-2), first discovered in Wuhan, China, in 2019, the rapidly growing number of infected people and casualties has posed a serious global threat [1]. SARS-CoV-2 causes severe respiratory symptoms that are accompanied by high fever, cough, and severe pneumonia [2], and although the mortality rate has been reported to be lower than that of severe acute respiratory syndrome coronavirus (SARS-CoV) or Middle East respiratory syndrome coronavirus (MERS-CoV), the overall risk remains highly significant, and thus novel prophylactic agents such as therapeutic drugs and vaccines are urgently in need.

Among the four coronavirus genera (α, β, γ, and δ), SARS-CoV-2 belongs to the β-coronaviruses and is an enveloped, positive-sense single-stranded RNA (or (+) ssRNA) virus, the RNA genome of which encodes structural proteins including the spike (S) protein [3,4]. SARS-CoV-2 shares similarities in its genome sequences with those of SARS-CoV and MERS-CoV, which are respectively about 79.5% and 50% similar, indicating homologous structures and similar infectious pathways to SARS-CoV [5].

As in all coronaviruses, the S protein is present on the surface of the virus and plays a critical role in the viral entry to host cells [6,7]. The S protein consists of two subunits, S1 and S2, which are non-covalently associated as a homotrimeric form that comprises a prefusion state. The receptor-binding domain (RBD, residues 387–516) of the S1 subunit consists of a core domain and a receptor-binding motif (RBM, residues 438–505), and this motif directly engages with the host receptor, known as angiotensin-converting enzyme 2 (ACE2) [8]. Upon entry of the virus into cells, the RBD of the S1 subunit recognizes ACE2 on the surface of host cells as a receptor, while the S2 subunit has a role in viral fusion with host cell membranes and is primed by the S protein cleavage at the S1/S2 and S2′ sites on the S2 subunit through intracellular proteases such as TMPRSS2, triggering the conformational change of the S protein to the postfusion state [8,9,10,11,12,13].

Due to the urgent situation in which no drugs or vaccines are available, researchers and medical doctors have worked in close cooperation to develop a variety of therapeutic approaches, mostly repurposed from existing drugs, including nucleoside analogs such as remdesivir [14,15,16], antiparasitics such as chloroquine [17], protease inhibitors such as lopinavir and ritonavir [18], indole-derivate molecules such as arbidol [19], plasma therapy from convalescent patients who recovered from the infection [20], and, lastly, antibodies that treat the viral infection by blocking the S protein or pro-inflammatory cytokines, such as IL-6, TNF-α, GM-CSF, and IFN-γ [21,22].

Monoclonal antibodies (mAbs) have been recognized as significant biologics in therapeutic fields and are now rapidly taking a position as an alternative treatment that complements vaccines in working against newly emerging pathogenic viruses, such as SARS-CoV-2 [22,23,24,25]. Since viral neutralization by targeting the S protein has previously been shown to correlate with therapeutic efficacy in animal models [26], tremendous efforts have been made, based on the structural information of the S protein and its critical role in viral entry, to discover neutralizing mAbs that block the RBD of the S1 subunit through a variety of approaches, such as phage display library selection [27,28,29,30]; antibody selection through immunization of animals, such as humanized mice, dromedary camels, or sharks [31,32,33,34]; and antibody isolation from memory or plasma B cells of naturally infected human donors [31,35,36,37,38,39]. At the time of writing, 198 antibodies programs are in discovery and development phases globally, among which 66 are going through clinical trials (phase 1/2/3). In particular, 122 antibodies programs (~62%) are known to target the S protein of SARS-CoV-2, highlighting the importance of the S protein as a target. Last November, the United States Food and Drug Administration (US FDA) approved two antibodies targeting the S protein (REGN-COV2 (REGN10933 + REGN10987) and Bamlanivimab from Regeneron and Eli Lilly, respectively) for the treatment of COVID-19 patients [21].

However, the mAb approach, mainly based on the immunoglobulin G (IgG) format, has a drawback in that it relies on mammalian cell lines for the production of antibodies, which is costly and time-consuming. Moreover, viruses can easily evolve to generate RBD variants with mutations avoiding immune responses [39]. Therefore, in order to protect against the immune escapers, it would be useful to identify a selection of mAbs broadly acting on different epitopes that could contribute to a therapeutic antibody cocktail that might induce resistance to mutations in the RBD variants [29,40,41]. 

In this study, we panned a human Fab synthetic phage display library on the RBD of the SARS-CoV-2 S protein and obtained human mAbs with desirable properties that successfully neutralized the viral entry upon SARS-CoV-2 infection. We anticipate that these mAbs can be further developed as a promising antibody therapy against the pathogenic virus and as tools for diagnosis.

## 2. Results

### 2.1. Selection of Human Anti-SARS-2 RBD Fabs

**Figure 1:**
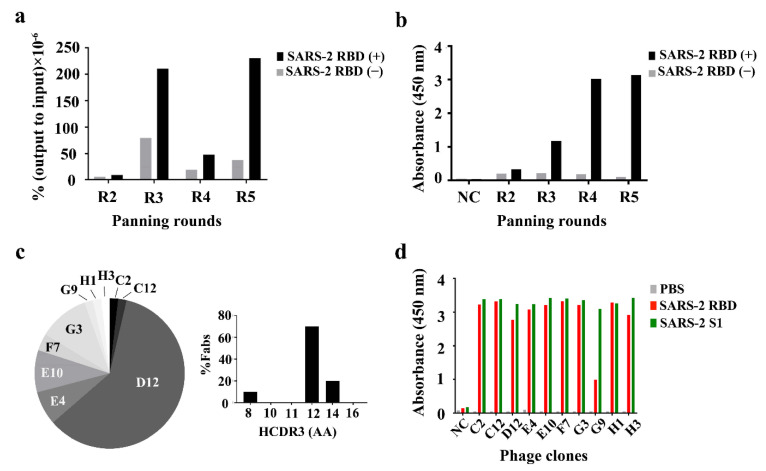
Panning of the phage-displayed synthetic Fab library on an immobilized SARS-2 receptor-binding domain (RBD). (**a**) Monitoring of the phage titers over four rounds (R2–R5) of panning. Black and gray bars indicate the ratio of the phage output to the input titers, presented as a percentage (%), from panning on immobilized SARS-2 RBD (black, SARS-2 RBD (+)) and non-immobilized SARS-2 RBD (gray, SARS-2 RBD (−)) surfaces. The ratio of the output to the input (%) = (phage output titer ÷ phage input titer) × 100. (**b**) Phage ELISA performed on the immobilized SARS-2 RBD surfaces using each panning library phage. (**c**) Frequency of ten Fab phage clones selected in the third and fourth rounds (left) and the distribution of HCDR3 lengths (right). The selection frequency of a unique clone (%) = (number of unique clones ÷ total number of phage ELISA positives) × 100. (**d**) Monoclonal ELISA of ten Fab phage clones against the SARS-2 RBD (red) and SARS-2 S1 protein (green). AA: amino acid residue; NC: negative control.

To isolate a specific SARS-CoV-2 antibody, the KFab-I library [42,43] was panned against a recombinant SARS-2 RBD immobilized on magnetic beads (Figure 1a). After five rounds of panning, the phage ELISA was performed on immobilized SARS-2 RBD surfaces, using each panning library to monitor the enrichment (Figure 1b). Ninety-five monoclonal phages were randomly picked from the third and fourth rounds, and the binding on the SARS-2 RBD was evaluated by ELISA (Figure S1). Of the 190 individual clones from the third and fourth rounds, 70 clones showed higher absorbances at 450 nm (A450 nm) than those of the negative control (no immobilized SARS-2 RBD control) in the ELISA read-out. The 70 clones were sequenced: 55 were confirmed to be complete and the remaining clones had mutations, such as frame-shifts and stop codons. By analyzing the CDR sequences of the 55 clones, ten unique Fab clones were identified in total and, of these, D12 (Fab) was dominantly selected (60% of sequenced clones (33 out of the 55)), while the other clones showed selection frequencies of about 11% (G3 (Fab)), 9% (E10 (Fab)), 7% (E4 (Fab)), 4% (F7 (Fab)), and 2% (C2 (Fab), C12 (Fab), G9 (Fab), H1 (Fab), and H3 (Fab)) (Figure 1c). In addition, it was observed that nine out of the ten Fabs had CDR3 lengths greater than 12 amino acid residues (70% and 20% for 12 and 14 amino acid residues, respectively), while 10% of the Fabs had shorter CDR3 lengths, such as eight amino acid residues (Figure 1c). In order to confirm that the ten selected clones bound to the SARS-2 RBD and SARS-2 S1 proteins as well, a binding assay was performed, revealing that all of the antibody clones bound to the RBD and the S1 proteins (Figure 1d). In parallel, a binding analysis for RBD variants was also performed using the ten selected clones (Figure S2). This showed that all the phage clones bound to each RBD variant. Two of them (G9 and E4) bound weakly to one RBD variant (N354D; D364Y).

### 2.2. Production and Characterization of Human Anti-SARS-2 RBD Fabs

To order to produce and characterize the selected clones as Fab proteins, clones were cloned into an in-house *E. coli* expression vector (pKFAB). The Fab proteins were expressed and purified as described in the Section 4. The protein yields of these Fab clones were 11 mg/L, 6.5 mg/L, 106 mg/L, 15.5 mg/L, 12.5 mg/L, 125.5 mg/L, 8.5 mg/L, 17.5 mg/L, 9.5 mg/L, and 40 mg/L for C2 (Fab), C12 (Fab), D12 (Fab), F7 (Fab), H1 (Fab), E4 (Fab), E10 (Fab), G3 (Fab), G9 (Fab), and H3 (Fab), respectively (Figure S3 and Table 1).

**Figure 2:**
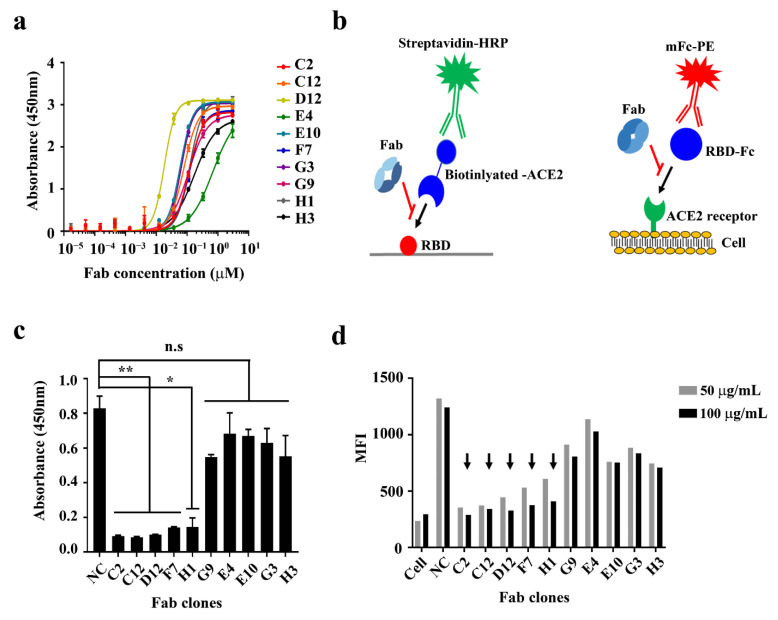
Characterization of human anti-SARS-2 RBD Fabs. (**a**) Soluble ELISA of ten serially diluted human anti-SARS-2 RBD Fabs on immobilized SARS-2 RBD surfaces to measure their apparent affinities (*EC*_50_, nM). (**b**) Schematic drawings of a competitive ELISA of human anti-SARS-2 RBD Fabs between the SARS-2 RBD and ACE2 protein (left) or ACE2-overexpressed cells (right). (**c**) Competitive ELISA of human anti-SARS-2 RBD Fabs antagonizing the interaction between ACE2 and the SARS-CoV-2 RBD. (**d**) Competitive flow cytometry analysis of human anti-SARS-2 RBD Fabs antagonizing the interaction between ACE2 on cells and the SARS-CoV-2 RBD (tagged with mouse Fc (mFc)). Arrows indicate potentially neutralizing clones. mFc-PE: anti-mouse PE (phycoerythrin) conjugate; MFI: mean fluorescence intensity; n.s: not significant (*p* > 0.05); NC: negative control. * and **: *p* < 0.05 and *p* < 0.01, respectively.

The apparent affinities of the ten Fabs for the SARS-2 RBD were assessed using an ELISA (*EC*_50_, nM) (Figure 2a and Table 1). While six Fabs (G9 (Fab), C2 (Fab), F7 (Fab), H1 (Fab), H3 (Fab), and E4 (Fab)) had low to intermediate apparent affinities for the SARS-2 RBD (*EC*_50_ = 112–663 nM), the remaining four Fabs—D12 (Fab), E10 (Fab), G3 (Fab), and C12 (Fab)—showed relatively higher apparent affinities (19 nM, 62 nM, 67 nM, and 83 nM, respectively) (Figure 2a).

To examine the potential neutralizing ability of the selected Fabs, we conducted a competitive binding assay between the SARS-2 RBD and ACE2 protein or ACE2-overexpressed cells (Figure 2b). It was found that five Fabs (C2 (Fab), C12 (Fab), D12 (Fab), F7 (Fab), and H1 (Fab)) significantly antagonized the interaction between the SARS-2 RBD and biotinylated ACE2 protein (Figure 2c). The same five Fabs seemed to block the interaction between SARS-2 RBD-mFc protein and ACE2-overexpressed cells in a flow cytometry analysis as well (Figure 2d and Figure S4).

Next, in order to determine whether the ten Fabs could cross-react with the S1 proteins from other coronaviruses, such as SARS-CoV and MERS-CoV, an ELISA was conducted. This showed that three of the Fabs (E4 (Fab), E10 (Fab), and G3 (Fab)) indeed cross-reacted with the SARS-CoV S1, whereas no Fabs bound with the MERS-CoV S1 (Figure S5).

### 2.3. Production and Characterization of Human Anti-SARS-2 RBD IgGs

To produce and characterize the five anti-SARS-2 RBD antibodies that seemed to have neutralizing activities in IgG forms, the five Fabs were individually reformatted to IgG forms. That is, the individual VH and VL sequences from each of the Fabs were cloned into heavy- (IgG1 Fc) and light-chain (Ck1) expression vectors, respectively. The five IgGs were transiently expressed in HEK293 cells and subsequently purified as described in the Section 4. The resulting IgGs were highly pure and their protein yields were 9.6 mg/L, 12.9 mg/L, 13.5 mg/L, 13.2 mg/L, and 12.5 mg/L for C2 (IgG), C12 (IgG), D12 (IgG), F7 (IgG), and H1 (IgG), respectively (Figure S6 and Table 1).

**Figure 3:**
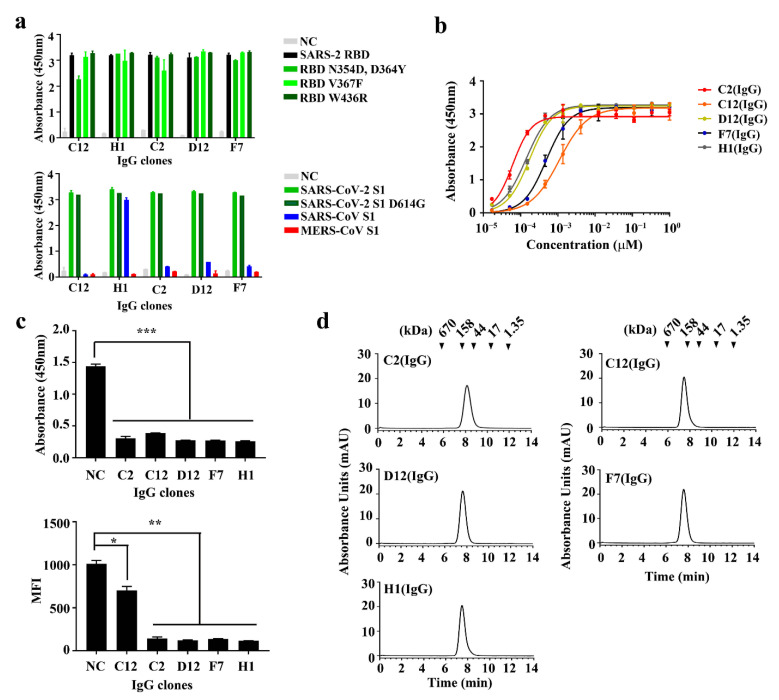
Characterization of anti-SARS-2 RBD immunoglobulin Gs (IgGs). (**a**) Binding analysis of five human anti-SARS-2 RBD IgGs—C12 (IgG), H1 (IgG), C2 (IgG), D12 (IgG), and F7 (IgG)—to the SARS-2 RBD and its variants (top) and the SARS-CoV-2 S1 (D614G) and other coronavirus S1 proteins (bottom), respectively. (**b**) Soluble ELISA of five serially diluted human anti-SARS-2 RBD IgGs on immobilized SARS-2 RBD surfaces to measure their apparent affinities (*EC*_50_, nM). (**c**) ELISA detection for five human anti-SARS-2 RBD IgGs blocking the binding of the ACE2 protein with the SARS-CoV-2 RBD (top) and analysis of the flow cytometry for the blocking effect between the SARS-CoV-2 RBD and an ACE2-overexpressed cell (bottom). (**d**) Size-exclusion chromatography analysis of five human anti-SARS-2 RBD IgGs. The positions of the molecular mass markers, shown as kDa, on the retention time *x*-axis are indicated above the peaks. The data are presented as the mean ± standard error (SEM). MFI: mean fluorescence intensity; NC: negative control; *, **, and ***: *p* < 0.05, *p* < 0.01, and *p* < 0.001, respectively.

In order to confirm whether the purified IgGs could bind to the SARS-2 RBD and its variants—and also whether they could cross-react with other coronavirus S1 proteins, as observed with the Fabs—an ELISA binding assay was conducted, revealing that all the IgGs bound to the SARS-2 RBD and SARS-2 S1, as well as the RBD variants, whereas all of them did not bind to the MERS-CoV S1 (Figure 3a). In particular, one clone, H1 (IgG), was found to cross-react with the SARS-CoV S1 and three IgGs (C2 (IgG), D12 (IgG), and F7 (IgG)) seemed to bind with the SARS-CoV S1 but the binding was too weak to confirm their cross-reactivity.

To determine whether the apparent affinities of the anti-SARS-2 RBD IgGs were altered by reformatting the Fabs into the IgGs, the apparent affinities of the IgGs were examined using ELISA (*EC*_50_, nM). As shown in Figure 3b, the five clones (C2 (IgG), C12 (IgG), D12 (IgG), F7 (IgG), and H1 (IgG)) increased their apparent affinities approximately 100- to 1800-fold compared to their Fab formats (Figure 3b and Table 1), which might have been due to an avidity effect [42,43]. Next, a size-exclusion chromatography analysis was performed to assess their non-aggregation properties, revealing that the IgGs were monomeric without forming high molecular weight (HMW) aggregates (Figure 3d and Table 1). The five IgGs were further analyzed using a protein thermal shift (PTS) assay to determine their thermal stabilities; the assay showed that all the IgGs had *T*_m_ over 70.0 °C, confirming that they were thermally stable (Figure S7 and Table 1). To determine whether the thermal stability of the IgGs was due to the intrinsically high stability of the Fabs, the five Fabs were analyzed with the same PTS assay and the results showed that all of the Fabs had *T*_m_ values over 76.0 °C, indicating that the high thermal stability of the IgGs was derived from the intrinsic properties of the Fabs (Figure S8).

Next, to determine whether the neutralizing activities of the SARS-2 RBD IgGs remained after reformatting the Fabs into IgGs, we performed a competitive binding assay demonstrating the IgGs’ antagonizing activities in the interaction between the SARS-2 RBD and ACE2 protein or ACE2-expressed cells (Figure 3c). All the IgGs significantly antagonized the interaction between the RBD and biotinylated ACE2 protein (Figure 3c top) and also inhibited the interaction between the SARS-2 RBD-mFc protein and ACE2-overexpressed cells in a flow cytometry analysis, although C12 (IgG) showed a slightly reduced inhibition compared to the other IgGs (Figure 3c bottom and Figure S9).

### 2.4. Neutralization Assay against SARS-CoV-2 Pseudovirus and Authentic SARS-CoV-2

**Figure 4:**
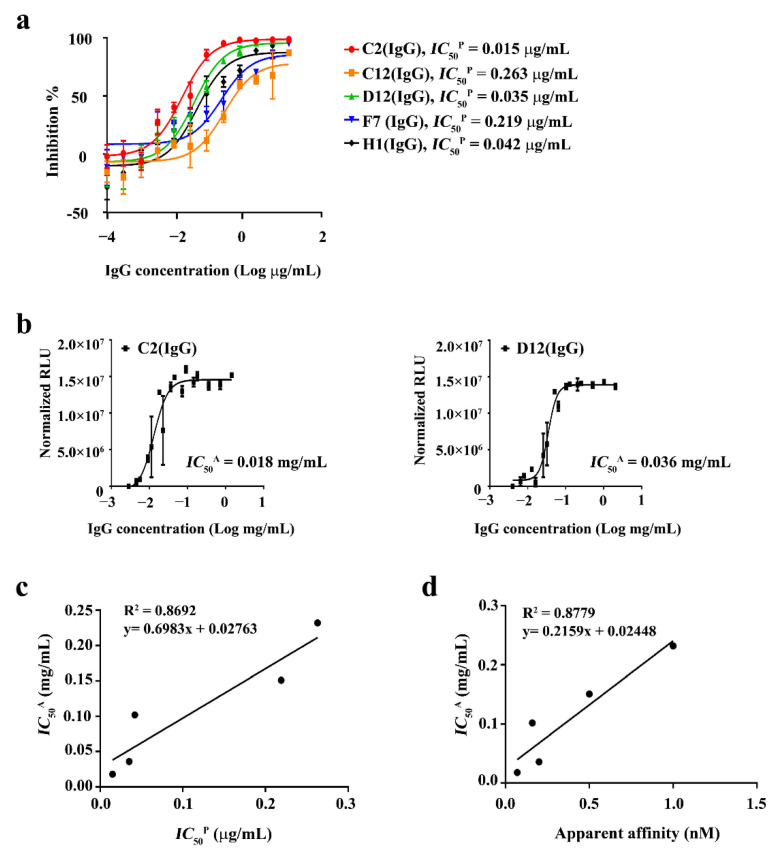
In vitro neutralization assay of human anti-SARS-2 RBD IgGs. Pseudo-typed virus-based neutralization (**a**) and a neutralization assay using authentic SARS-CoV-2 (**b**). (**c**) Correlation in neutralization potencies between pseudo-typed virus- and authentic virus-based assays. (**d**) Correlation between affinities of anti-SARS-2 RBD IgGs and their neutralization potencies for the authentic virus. The data are showed as the mean ± standard error (SEM).

To evaluate the neutralization potency of the five human SARS-CoV-2 RBD IgGs, we carried out a pseudo-typed virus neutralization assay using a lentiviral HIV-1 pseudo-typing system [44]. The five IgGs were found to display strong neutralizing activity against the SARS-CoV-2 pseudo-typed virus, among which C2 (IgG) and D12 (IgG) showed the most potent activity. The *IC*_50_ values of C2 (IgG) and D12 (IgG) in the pseudo-typed virus neutralization were 0.015 and 0.035 μg/mL, respectively (Figure 4a and Table 1).

Based on our previous competitive binding assays, we examined the five IgGs in order to evaluate their neutralizing effects on authentic SARS-CoV-2. The observations of luminescent signals showed that two of the IgGs, C2 (IgG) and D12 (IgG), exhibited high protection upon SARS-CoV-2 exposure for three days. The *IC_50_* values of C2 (IgG) and D12 (IgG) in the authentic SARS-CoV-2 neutralization were 0.018 and 0.036 mg/mL, respectively (Figure 4b, Figure S10, and Table 1). The rest of the IgGs showed less neutralization compared to the two IgGs: 0.102 mg/mL, 0.151 mg/mL, and 0.232 mg/mL for H1 (IgG), F7 (IgG), and C12 (IgG), respectively (Table 1). In order to know whether there was any correlation present between the neutralization potencies from the pseudo-typed virus and the authentic virus, we compared and plotted the values from the assays and found that a strong correlation was indeed present between the neutralization potencies from the two different neutralization assays (Figure 4c). In addition, we also found that there was a strong correlation between the affinity and the neutralization potency of the anti-SARS-2 RBD IgGs as well (Figure 4d).

The two human anti-SARS-2 RBD IgGs, C2 and D12, were further characterized by BLI (Octet) in order to determine their affinities with the SARS-2 RBD, and it was found that C2 (IgG) and D12 (IgG) had binding affinities of 0.13 nM and 0.57 nM, respectively (Figure S11). This confirmed that some avidity effects were reflected in the apparent affinities from the previous ELISA (Figure 3b and Table 1), which was performed with an immobilized SARS-2 RBD, unlike the BLI (Octet), which was undertaken with the human IgGs immobilized on the sensor. 

## 3. Discussion

We here report on the selection of human mAbs specific to the SARS-CoV-2 RBD (SARS-2 RBD) using a human synthetic Fab phage display library. Phage display is a powerful tool that has been used for both discovery and therapeutic applications against various malignancies, including infectious diseases [45,46,47,48,49]. In particular, phage display has been demonstrated to be highly effective for the selection of human antibodies against SARS-CoV-2 in both synthetic and immune phage display libraries built with immune repertoires of memory or plasma B cells from convalescent patients who recovered from the viral infection [27,28,29,30]. In our phage display panning, we employed two in-house human synthetic Fab phage display libraries: KFab-I and KFab-II. The KFab-I library was built on V_H_3 and V_k_1 frameworks by randomizing their complementarity-determining regions (CDRs) and yielded ten human anti-SARS-2 RBD mAb clones, whereas no binder was yielded by panning with the KFab-II library, another in-house human synthetic Fab phage display library built on V_H_1 and V_k_1 frameworks. Due to the same CDR randomization design being applied to the two libraries, we reasoned that the framework could have made a difference in the panning outcome. The human V_H_3 family has been shown to have the highest stability and soluble protein yield, and its germline usage out of 51 germline genes is about 43%, which is considerably higher than that of other families of human V_H_ [43,50]. Indeed, for various antibody libraries, such as the Griffiths and the HuCAL libraries, it has been shown that a considerable number of antibodies selected from the libraries belonged to the human V_H_3 family (74% and 36% for the Griffiths and HuCAL libraries, respectively) [51,52], indicating that the human V_H_3 framework may be inevitably favored in the phage display selection due to its desirable properties. Moreover, our previous phage display panning against human YKL-40, which was also performed with the two Fab libraries, showed a similar outcome in that, unlike the KFab-I library, the KFab-II yielded no binders with desirable properties [43]. However, it is believed that a panning with a mixture of both V_H_3 and V_H_1 frameworks from KFab-I and KFab-II libraries might result in a different outcome from the previous panning performed separately with each Fab library.

Our study revealed that only one anti-SARS-2 RBD IgG clone (H1) cross-reacted with the S protein of SARS-CoV and the rest of the anti-SARS-2 RBD IgGs reacted specifically with the S protein of SARS-CoV-2, while no anti-SARS-2 RBD IgGs cross-reacted with the S protein of MERS-CoV, as expected based on the low protein sequence identity between SARS-CoV-2 and MERS-CoV [5,53]. The difference in the cross-reactivity of the antibodies between SARS-CoV and SARS-CoV-2 could be related to whether the epitopes recognized by antibodies are located on regions that are conserved between SARS-CoV-2 and SARS-CoV. The amino acid sequence identity of the RBD (residues 387–516) between SARS-CoV-2 and SARS-CoV is quite high (86.3%), whereas the sequence identity of the receptor-binding motif (RBM; residues 438–505) is substantially lower (46.7%) [8,22,32]. This suggests that our antibodies likely recognize epitopes on the RBM of each virus, not on the conserved regions of the RBD. In addition, the RBM is known to have a loop structure and is thus likely subjected to the conformational variation, which may further reduce the structural homology between SARS-CoV-2 and SARS-CoV [8,22]. In addition, we also observed that all of the anti-SARS-2 RBD IgGs cross-reacted with the SARS-2 RBD variants tested, indicating that the antibody epitopes might not have overlapped with the regions of the RBD in which mutations occurred or that the anti-SARS-2 RBD IgGs might have been tolerable enough to bind to the RBD variants, although the antibody epitopes overlapped with regions where mutations occurred. Due to the limited numbers of the variants tested, it is hard to tell how tolerable our antibodies are to the genetic variations. More information on the antibody epitopes and antibody binding against increased numbers of the RBD variants will surely elucidate this.

Although in general human mAbs targeting the RBD of the S1 subunit have higher neutralizing potencies than those targeting other regions of the S protein, such as the S2 subunit and other regions of the S1 (e.g., the N-terminal domain (NTD)), it is still necessary to combine human mAbs that recognize different neutralizing epitopes due to the emergence of viruses carrying RBD mutations. Indeed, this was nicely demonstrated by the Regeneron antibodies (REGN10933 + REGN10987): an antibody cocktail consisting of these two antibodies, which recognize distinct, non-overlapping epitopes on the RBD, helped to avoid escape mutants after treatment thanks to the unlikely occurrence of simultaneous mutations on two distinct genetic sites [40]. By the same rationale, the neutralizing antibody (4A8 mAb) targeting the NTD of the S1 subunit could also be a good candidate for antibody cocktail therapy [35]. We are currently working to figure out whether our neutralizing antibodies (C2 and D12) can compete with other neutralizing antibodies, such as REGN-COV2, by charactering their antibody epitopes and by using a competitive enzyme-linked immunosorbent assay (ELISA) or bio-layer interferometry (BLI).

In order to identify potential neutralizing antibody candidates out of the ten Fab clones from the panning, we used in vitro competitive assays, namely an ELISA and an ACE2-overexpressed cell-based assay, to enable the Fab clones to compete with either a biotinylated ACE2 or the SARS-2 RBD, respectively. The two assays led to the identification of five Fabs as potential neutralizing antibodies, with the five candidate Fab clones behaving similarly in both assays. When the same assays were performed with the same five candidate antibodies that were reformatted from Fabs to IgGs, the two assays confirmed the five IgG antibodies as competitors, although the clone C12 (IgG) showed slightly less competition, albeit still significant, in both assays, especially in the ACE2-overexpressed cell-based assay. Although the two in vitro assays we adopted were not sensitive enough to discern their subtle differences and the antibodies could therefore not be ranked, it was strongly demonstrated that they could still be useful to handle many clones when screening potential candidates prior to a virus-mediated neutralization assay for either a pseudo-typed or authentic virus.

In the characterization of the antibodies in terms of affinity and neutralization, we also noticed that the affinity of the antibodies seemed to correlate with the neutralization potency. That is, the order of the affinity, C2 > D12 > H1 > F7 > C12, strongly correlated with the order of the neutralization potency, C2 > D12 > H1 > F7 > C12. This observed correlation is strongly supported by previous studies: (1) in a study of the mAb IIB4 recognizing influenza A virus haemagglutinin (HA), a strong positive correlation between its affinity and viral neutralization was found [54]; (2) in a study with potential SARS-CoV-2 neutralizing antibodies from convalescent human patients, RBD binding and viral neutralization were well correlated [55].This therefore suggests that further maturation of the affinity of the mAbs may somehow enhance their neutralization potency accordingly; studies are underway to explore this. Moreover, the neutralization potencies determined by an in vitro neutralization assay for pseudo-typed and authentic SARS-CoV-2 also correlated with each other: the order of neutralization potency from the pseudo-typed virus, C2 > D12 > H1 > F7 > C12, nicely correlated with the order of potency from the authentic virus, C2 > D12 > F7 > H1 > C12, indicating that a neutralization assay for a pseudo-typed virus can be reliably applied to assess the neutralization potency of clones prior to the authentic virus-based assay, which, unlike the pseudo-typed viral assay, must be done under Biosafety Level 3 (BSL3) conditions. Consistent with the antibody binding against the D614G S1 variant, the order of neutralization potency for the pseudo-typed virus (carrying the D614G S1 variant) remained the same as the order for the pseudo-typed virus (carrying the D614 wildtype S1), with C2 (IgG) and C12 (IgG) showing the highest and the lowest neutralizations, respectively, thus confirming that the antibodies were tolerable to the D614G variation on the S1. This result highlights that current vaccinations relying on the neutralization of antibodies targeting the wildtype S protein of SARS-CoV-2 in vivo may somehow also be effective in coping with the new SARS-CoV-2 variants, including the D614G variant [56].

In conclusion, we selected human anti-SARS-2 RBD mAbs from a human synthetic Fab phage display library. We characterized the resulting Fabs and IgGs in order to observe their desirable biophysical properties, such as their affinity, non-aggregation, and thermal stability. We conducted in vitro assays to assess their neutralizing activities against pseudo-typed and authentic SARS-CoV-2 and identified two clones, C2 and D12, which demonstrated an exceptional ability to block the viral entry into cells. Further refinement of the mAbs should allow for the development of promising anti-SARS-CoV-2 therapeutics, as well as reagents for diagnosis.

## 4. Materials and Methods

### 4.1. A Phage Library Display Panning

Human synthetic Fab phage display libraries produced in-house (KFab-I and KFab-II, respectively built on human VH3/Vk1 and human VH1/Vk1 germline-based scaffolds, with randomized complementarity-determining regions) were used for the selection of specific binders against a SARS-CoV-2 spike protein (SARS-2 RBD) (Sino Biological, Cat. 40592-V08H, Beijing, China). The SARS-2 RBD was coupled to beads following the protocol for dynabeads (Thermofisher Scientific, Cat. 14301, Waltham, MA, USA). After removing the supernatant on the beads, the coated beads were blocked with 5% skimmed milk (BD, Cat. 232100, Franklin Lakes, NJ, USA) in PBS for 1 h at room temperature. At the same time, the phage library was incubated in 2% skimmed milk in PBS for 1 h at room temperature. The blocked phages were transferred to the beads coated with SARS-2 RBD and incubated for 2 h at room temperature. After separating the beads from the supernatant, the beads bound with phages were washed three times with PBST (PBS containing 0.05% Tween 20) and bound phages were eluted from the beads with 100 mM triethylamine (Sigma-Aldrich, Cat. 90335, St. Louis, MO, USA) for 10 min at room temperature, followed by neutralization with 1 M Tris-HCl (pH 7.4) (Biosesang, Cat. T2016-7.5, Seongnam, Korea). The eluted phages were used to infect *E. coli* TG1 cells at OD_600_ 0.6~0.8. Phage particles were prepared for subsequent rounds of panning by amplification and rescue using VCSM13 helper phages (provided by Dr. Hong from Kangwon National University, Chuncheon, Gangwon-do, Korea) according to standard procedures. The amplified phage was used for the next round of panning, and so forth.

### 4.2. Polyclonal Phage ELISA

A polyclonal phage ELISA was performed using pools of purified phage from each library stock. A 96-Well Half-Area Microplate (Corning, Cat. 3690, New York, NY, USA) was coated overnight at 4 °C, with 30 μL per well of 1 μg/mL SARS-2 RBD (Sino Biological, Cat. 40592-V08H, Beijing, China), and each well was blocked with 5% skimmed milk in PBS (MPBS) for 1 h at room temperature. Phage pools (~10^12^ phage particles) were also blocked in MPBS for 1 h at room temperature and then blocked phage pools were added to the SARS-2 RBD-coated plate and incubated for 1 h at 37 °C. After washing four times with PBST, the horseradish peroxidase (HRP)-conjugated anti-M13 antibody (1:5000, Sino Biological, Cat. 11973-MM05, Beijing, China) was incubated for 1 h at 37 °C. After washing four times with PBST, a TMB substrate solution (Sigma-Aldrich, Cat. T0440, St. Louis, MO, USA) was added for 8 min, and the reaction was stopped with 1 N sulfuric acid (Merck, Cat. 100731, Darmstadt, Germany). The absorbance was measured at 450 nm using a SpectraMax 190 Microplate Reader (Molecular Devices, Sunnydale, CA, USA).

### 4.3. Monoclonal Phage ELISA

Individual phage clones from either the third or fourth round were tested for binding to the SARS-2 RBD-coated plate. Several 96-Well Half-Area Microplates (Corning, Cat. 3690, New York, NY, USA) were coated overnight at 4 °C, with 30 μL per well of 1 μg/mL SARS-2 RBD, and each well was blocked with 5% skimmed milk in PBS for 1 h at room temperature. The amplified phages of individual clones from the third or fourth rounds of panning were added and incubated for 1 h at 37 °C. After washing four times with PBST, the horseradish peroxidase-conjugated anti-M13 antibody (1:5000, Sino Biological, Cat. 11973-MM05, Beijing, China) was incubated for 1 h at 37 °C. After washing four times with PBST, a TMB substrate solution (Sigma-Aldrich, Cat. T0440, St. Louis, MO, USA) was added for 8 min, and the reaction was stopped with 1 N sulfuric acid (Merck, Cat. 100731, Darmstadt, Germany). The absorbance was measured at 450 nm using a SpectraMax 190 Microplate Reader (Molecular Devices, Sunnydale, CA, USA).

### 4.4. Production of Fab Proteins

An in-house bacterial expression vector (pKFAB) was used to construct the Fab expression vectors. The Fab fragments and pKFAB vector were amplified by a polymerase chain reaction (PCR) for each primer set. The PCR products were treated with DpnI (New England Biolabs, Cat. R0176L, Ipswich, MA, USA) for 1 h at 37 °C, separated on a 1.2% agarose gel, and the single band was purified using a Wizard SV Gel and PCR Clean-Up System (New England Biolabs, Cat. A9282, Ipswich, MA, USA). The fragments were assembled following the Gibson assembly protocol (New England Biolabs, Cat. E2611, Ipswich, MA, USA). The assembled products were used to transform *E. coli* DH5α competent cells (Enzynomics, Cat. CP010, Daejeon, Korea). The individual colonies of the transformed cells were isolated and the sequences of the isolated clones were verified.

Top10F’ Competent Cells (Invitrogen, Cat. C303003, Carlsbad, CA, USA) were transformed with the Fab expression vectors and the transformants were grown in 200 mL of TB (Terrific Broth) (Thermofisher Scientific, Cat. 22711022, Waltham, MA, USA) media supplemented with 100 μg/mL ampicillin at 37 °C until the OD_600_ reached 0.5. The log-phase cultures were then induced with 0.5 mM isopropyl β-D-1-thiogalactopyranoside (IPTG) (DAWINBIO, Cat. I0355-005, Hanam, Gyeonggi, Seoul) and incubated overnight at 30 °C. The cells were collected and resuspended in 16 mL of 1× TES (50 mM Tris-HCl, 1 mM EDTA, 20% Sucrose, pH 8.0). After incubation for 30 min on ice, 24 mL of 0.2× TES was added and incubated for 1 h on ice. The periplasmic fractions were collected after centrifugation at 12,000 rpm for 30 min and filtered through a 0.22 μm filter (Milipore, Cat. SCGP00525, Carrigtwohill, Co., Cork, Ireland). The periplasmic extracts were loaded on a column packed with 0.5 mL of ProL (rProtein L) Agarose resin (Amicogen, Cat. 3010125, Jinju, Gyeongnam-do, Korea). The column was washed with 10 column volumes (CVs) of PBS and eluted with 30 CVs of Buffer W (100 mM Glycine, pH 2.5). The eluted proteins were neutralized with 1M Tris-HCl (pH 9.0) (Biosesang, Cat. TR2016-050-90, Seongnam, Gyeonggi, Korea). The eluted protein was concentrated and buffer-exchanged with PBS using Amicon Ultra-15 Centrifuge Filter Units (Milipore, Cat. UFC903024, Carrigtwohill, Co., Cork, Ireland).

### 4.5. Determination of Apparent Affinity by ELISA

Several 96-Well Half-Area Microplates (Corning, Cat. 3690, New York, NY, USA) were coated overnight at 4 °C, with 30 μL per well of 2 μg/mL SARS-2 RBD. After rinsing them twice with tap water, the wells were blocked with 5% skimmed milk in PBS for 1 h at room temperature. Serially diluted anti-SARS-2 RBD Fabs or IgGs were added and incubated for 1 h at 37 °C. After washing the plates four times with PBST, the HRP-conjugated human kappa light-chain antibody (1:5000, Bethyl laboratories, Cat. A80-115P, Montgomery, TX, USA) or HRP-conjugated human IgG Fc (1:5000, Abcam, Cat. ab97225, Cambridge, USA) were added to the plates and incubated at 37 °C for 1 h. After washing the plates four times with PBST, a TMB substrate solution was incubated for 8 min, and the reaction was stopped with 1 N sulfuric acid. The absorbance was measured at 450 nm using a SpectraMax 190 Microplate Reader. A plot was created using a nonlinear regression with Graphpad Prism 7 (GraphPad Software, San Diego, CA, USA), and half-maximal effective concentration (*EC_50_*) values were determined accordingly.

### 4.6. ELISA-Based Neutralizing Assay

A 96-Well Half-Area Microplate (Corning, Cat. 3690, New York, NY, USA) was coated overnight at 4 °C, with 30 μL per well of 2 μg/mL SARS-2 RBD. After rinsing them twice with tap water, the wells were blocked with 5% skimmed milk in PBS for 1 h at room temperature. Both anti-SARS-2 RBD Fabs or IgGs and biotinylated human ACE2 (Acrobiosystems, Cat. AC2-H82E6, Newark, NJ, USA) were added and incubated for 1 h at 37 °C. After washing the plates four times with PBST, High Sensitivity Streptavidin-HRP (1:5000, Thermofisher, Cat. 21130, Waltham, MA, USA) was added to the plates and incubated for 1 h at 37 °C. After washing the plates four times with PBST, a TMB substrate solution was incubated for 8 min, and the reaction was stopped with 1 N sulfuric acid. The absorbance was measured at 450 nm using a SpectraMax 190 Microplate Reader. Graphpad Prism 7 (GraphPad Software, San Diego, CA, USA) was used to plot data using a two-way ANOVA algorithm.

### 4.7. Flow Cytometry-Based Neutralizing Assay

Calu-3 cells were obtained from the American Type Culture Collection (Manassas, VA, USA). Calu-3 cells were seeded in a 96-well plate (Corning, Cat. 3894, New York, NY, USA) at a density 1 × 10^6^ cells per well. Afterward, 50 μg/mL, 100 μg/mL Fabs, or 50 μg/mL IgGs were mixed with 5 μg/mL SARS-2 RBD-mFc (mouse IgG2a Fc-tagged SARS-2 RBD) (Acrobiosystems, Cat. SPD-C5259, Newark, NJ, USA), and the mixture was then incubated with cells for 1 h at 4 °C. After washing, the cell was labeled with the PE anti-mouse IgG2a antibody (Biolegend, Cat. 407108, San Diego, CA, USA) and incubated for 1 h at 4 °C. The cells were analyzed by FACS canto II (BD Biosciences, San Jose, CA, USA). Data were analyzed by FlowJo (downloadable at https://www.flowjo.com/solutions/flowjo/downloads (accessed on 1 January 2021)).

### 4.8. Conversion to IgG and Production of IgG Proteins

The light- and heavy-chain vectors (pcDNA3.4) were used as the backbone vectors. The VL and VH genes were individually amplified by polymerase chain reaction (PCR) from each Fab. The PCR products (VL and VH) were purified with an Expin PCR SV Mini Kit (Geneall, Cat. 103-102, Seoul, Korea) and digested with the following restriction enzymes (New England Biolabs, Ipswich, MA, USA): for VH, EcoRI (Cat. R3101S) and NheI (Cat. R3131S); for VL, XhoI (Cat. R0146S) and BsiWI (Cat. R3553S). The digestion products were separated on a 1.2% agarose gel, and the single band was purified with an Expin Gel SV Kit (Geneall, Cat. 102-102, Seoul, Korea). The fragments were ligated with the same restriction enzyme-digested vector using T4 DNA ligase (Promega, Cat. M1801, Madison, WI, USA). The ligation mixtures were used to transform *E. coli* DH5α Competent Cells (Enzynomics, Cat. CP010, Daejeon, Korea). Individual colonies of the transformed cells were isolated and the sequences of selected clones were confirmed by sequencing.

Freestyle 293 cells were cultured in Freestyle 293 Expression Medium (Thermofisher Scientific, Cat. 12338018, Waltham, MA, USA) in a humidified 8% CO_2_ incubator at 37 °C and 125 rpm. On the day of transfection, Freestyle 293 cell density was approximately 2.0 × 10^6^ cells/mL. Cells were transfected with plasmid DNA and these were mixed by DNA/PEI (polyethylenimine, Sigma-Aldrich, Cat. 913375, St. Louis, MO, USA) in a 1:2 ratio in the medium. Culture supernatants were collected after five days by centrifugation and filtration (0.22 µm, Polyethersulfone, Milipore, Cat. SLGPR33RB, Burlington, MA, USA).

Antibodies were purified from the culture supernatants using HiTrap MabSelect SuRe (GE Healthcare, Cat. 11-0034-94, Chicago, IL, USA) columns. Briefly, equilibration was carried out using Buffer A (1xPBS). The sample was loaded onto the equilibrated column. Following the sample loading, the column was washed with Buffer A until a stable baseline was established. Following the wash step, the protein was eluted with Buffer B (IgG elution buffer or 100 mM citrate buffer, pH 3.0). Following the elution, the IgG was brought to neutral pH with 1 M Tris base, pH 9.0, and dialyzed into a final buffer composition of PBS (pH 7.4) (Thermofisher Scientific, Cat. 10010023, Waltham, MA, USA). Each antibody was separated on 4–12% Bis-Tris gels (Thermofisher Scientific, Cat. NP0321, Waltham, MA, USA) with reducing or non-reducing conditions and stained with Sun-Gel Staining Solution (LPS Solution, Cat. SGS01, Daejeon, Korea).

### 4.9. Size-Exclusion Chromatography

The separation of the IgGs using size-exclusion chromatography (SEC) was performed using a Waters Alliance 2695 (Waters, Milford, MA, USA) connected to a Biosuite high-resolution SEC column (7.5 mm × 300 mm, 10 µm particle size, Waters, Milford, MA, USA). The separation was conducted using an isocratic elution with PBS, pH 7.4, at a flow rate of 1 mL/min. The effluent detection was conducted using a UV/Vis detector 2489 at 280 nm.

### 4.10. Determination of Melting Temperature by a Protein Thermal Shift (PTS) Assay

To each well of a MicroAmp Fast Optical 96-Well Reaction Plate (Applied Biosystems, Cat. 4346906, Foster City, CA, USA), 12.5 μL of anti-SARS-2 RBD Fabs or anti-SARS-2 RBD IgGs, 5 μL of Protein Thermal Shift Buffer and 2.5 μL of Protein Thermal Shift Dye (10×, Applied Biosystems, Cat. 4461146, Foster City, CA, USA) were mixed. As a negative control, PBS was mixed with the Protein Thermal Shift Dye. The plate was sealed with a MicroAmp Optical Adhesive Film (Applied Biosystems, Cat. 4306311, Foster City, CA, USA) and centrifuged at 1000 rpm for 1 min. The measurement was conducted using a real-time PCR instrument (ViiA 7 Real-Time PCR System, Thermofisher Scientific, Waltham, MA, USA). The instrument was set up according to the manufacturer’s instructions. All the experiments were performed at least in triplicate.

### 4.11. Production of SARS-CoV-2 Spike pseudovirus

Plasmids encoding the SARS-CoV-2 spike protein (D614) were purchased from Sino Biological (pCMV3-SARS-CoV-2 Spike, Cat. VG40589-UT, Beijing, China). The SARS-CoV-2 spike protein (D614G) was made by site-directed mutagenesis. The mutation was confirmed by full-length spike gene sequencing. The SARS-CoV-2 pseudoviruses were produced by co-transfection HEK-293T cells with pMDLg/pRRE (Addgene plasmid, 12251), pRSV-Rev (Addgene plasmid, 12253), pCDH-CMV-Nluc-copGFP-Puro (Addgene plasmid, 73037), and plasmids encoding either SARS-CoV-2 spike (D614) or SARS-CoV-2 spike (D614G) by using polyetherimide. Sixty hours post-infection, SARS-CoV-2 spike pseudoviruses containing culture supernatants were harvested, filtered (0.45 μm pore size, Millipore, Cat. S2HVU01RE, Burlington, MA, USA), and stored at −80 °C in 1 mL aliquots until use.

### 4.12. Pseudovirus Neutralization Assay

Derivatives of HEK-293T cells expressing ACE2 were generated by transducing HEK-293T cells with ACE2 (Addgene plasmid, 145839). Cells were used as single cell clones derived by limiting dilution from the bulk populations. The HEK-293T cells expressing ACE2 were seeded at a density of 1.5 × 10^4^ cells/well in 96-well luminometer-compatible tissue culture plates (Corning, Cat. 3610, New York, NY, USA) 24 h before infection. For the neutralization assay, 30 uL of pseudoviruses (~1 × 10^6^ RLU) was incubated with serial dilutions of the test antibody (12 dilutions in a threefold stepwise manner) for 1 h at 37 °C, together with the virus control, and then added to the 96-well 293T-ACE2 cells. After 24 h of incubation, the inoculum was replaced with fresh medium. Luciferase activity was measured 72 h after infection. Briefly, cells were washed twice, carefully, with PBS and lysed with 40 μL/well of a Passive Lysis buffer (Promega, Cat. E1941, Madison, WI, USA). Luciferase activity in lysates was measured using the Nano-Glo Luciferase Assay System (Promega, Cat. N1130, Madison, WI, USA). Specifically, 40 μL of the substrate in a Nano-Glo buffer was mixed with 40 μL of cell lysate and incubated for 3 min at RT. NanoLuc luciferase activity was measured using a Filter max F5 (Molecular Devices, San Jose, CA, USA) with an integration time of 1000 ms. The IC_50_ values were calculated with nonlinear regression using GraphPad Prism 7 (GraphPad Software, Inc., San Diego, CA, USA).

### 4.13. Authentic Virus Neutralization Assay

The SARS-CoV-2 virus (NCCP43326) for this study was provided by the National Culture Collection for Pathogens (Osong Health Technology Administration Complex, Cheongju, Chungbuk-do, Korea). Vero cells were seeded in a 96-well plate (Greiner Bio-One, Cat. 655180, Kremsmünster, Austria) at a density of 1 × 10^4^ cells per well. Serially, twofold-diluted mAbs and 100TCID_50_ (median tissue culture infectious dose) SARS-CoV-2 virus were incubated at RT for 0.5 h. mAb–virus mixtures were added to the Vero cells and incubated at 37 °C for 72 h. After 72 h of incubation, the supernatant was replaced with 100 μL of CellTiter-Glo^®^ 2.0 Reagent (Promega, Cat. G9241, Madison, WI, USA) and incubated at RT for 10 min. Luciferase activity in lysates was measured using the CellTiter-Glo^®^ 2.0 Assay (Promega, Cat. G9241, Madison, WI, USA). The luminescent signal was measured using a GloMax^®^ Discover Microplate Reader (Promega, Cat. GM3000, Madison, WI, USA) and the IC_50_ values were calculated by nonlinear regression using GraphPad Prism 7 (GraphPad Software, Inc., San Diego, CA, USA). This experiment was conducted at Chungbuk National University in a BSL3 facility (KCDC (Korea Center for Disease Control)-14-3-07).

### 4.14. Measurements of Affinity Using Bio-Layer Interferometry

Affinity measurement was performed by BLI using an Octet QK384 (ForteBio, Menlo Park, CA, USA) instrument. The anti-SARS-2 RBD human antibody was immobilized at 15 µg/mL in 10× Kinetic Buffer (KB) (ForteBio, Cat. 18-1105, Menlo Park, CA, USA). SARS-2 RBD protein was prepared in six different concentrations (100~0 nM, in twofold serial dilutions) in 10× KB for baseline stabilization. Before the binding measurements, the Anti-Human IgG Fc Capture (AHC; Cat. 18-5060) (ForteBio, Menlo Park, CA, USA) sensor tips were washed with 10× KB for 60 s and incubated in a binding buffer for 300 s (loading step). After a 180 s baseline dip in the same buffer, the binding kinetics were measured by dipping each human IgG (C2 and D12)-coated sensor into a well containing SARS-CoV-2 RBD protein at the above six concentrations. The binding interactions were monitored over a 300 s association step, followed by a 500 s dissociation step, in which the sensors were dipped into new wells containing 10× KB only. Non-specific binding was assessed using sensor tips without human IgGs. Data analysis was performed using Octet Data Analysis Software v6.4 (ForteBio, Menlo Park, CA, USA). Data were fitted to a 1:1 binding model to determine an association rate (K_on_, M^−1^s^−1^) and a dissociation rate (K_off_, s^−1^), and the equilibrium dissociation constant (K_D_) was calculated using the kinetic constants as follows: equilibrium dissociation constant (K_D_, M) = K_off_ ÷ K_on_.

### 4.15. Statistical Analysis

Statistical analysis was carried out using GraphPad Prism version 7.0 (GraphPad Software, San Diego, CA, USA). All error bars reported are the standard error of the mean (± SEM), unless otherwise indicated. Pairwise comparisons were conducted using an unpaired *t*-test. Differences between groups were considered significant at *p*-values below 0.05 (* *p* < 0.05; ** *p* < 0.01; *** *p* < 0.001).

## 5. Conclusions

We selected human anti-SARS-2 RBD mAbs from human synthetic Fab phage display libraries. We characterized the resulting Fabs and IgGs to observe their desirable biophysical properties, such as their high affinity, non-aggregation, and thermal stability. We conducted in vitro assays to assess their neutralizing activities against pseudo-typed and authentic SARS-CoV-2 and identified two clones, C2 and D12, which demonstrated an exceptional ability to block the viral entry into cells. Further refinement of the mAbs should allow for the development of promising human anti-SARS-CoV-2 therapeutic and diagnostic reagents.

## 6. Patents

We are in the process of obtaining a patent for the data on the human anti-SARS-2 RBD Fabs and IgGs in Korea (patent application number 10-2020-0161180; application date 26th November 2020).

## Figures and Tables

**Table 1:** Physicochemical properties of human anti-SARS-2 RBD antibodies.

Clones	Yield (mg/L Culture)	*T*_m_1/*T*_m_2 (°C)	Monomericity (Mon./Agg.)	*EC*_50_ (nM)	*K*_D_ (nM)	*IC*_50_^P^ (µg/mL)	*IC*_50_^A^ (mg/mL)
C2 (Fab)	11	80.2	n.d.	121	n.d.	n.d.	n.d.
C12 (Fab)	6.5	76.7/83.2	n.d.	83	n.d.	n.d.	n.d.
D12 (Fab)	106	76.2/83.0	n.d.	19	n.d.	n.d.	n.d.
F7 (Fab)	15.5	76.4	n.d.	125	n.d.	n.d.	n.d.
H1 (Fab)	12.5	76.8	n.d.	126	n.d.	n.d.	n.d.
E4 (Fab)	125.5	n.d.	n.d.	663	n.d.	n.d.	n.d.
E10 (Fab)	8.5	n.d.	n.d.	62	n.d.	n.d.	n.d.
G3 (Fab)	17.5	n.d.	n.d.	67	n.d.	n.d.	n.d.
G9 (Fab)	9.5	n.d.	n.d.	112	n.d.	n.d.	n.d.
H3 (Fab)	40	n.d.	n.d.	174	n.d.	n.d.	n.d.
C2 (IgG)	9.6	70.3/89.6	Mon.	0.07	0.134	0.015	0.018
C12 (IgG)	12.9	70.3/86.5	Mon.	1.0	n.d.	0.263	0.232
D12 (IgG)	13.5	70.5/91.9	Mon.	0.2	0.57	0.035	0.036
F7 (IgG)	13.2	70.2/81.1	Mon.	0.5	n.d.	0.219	0.151
H1 (IgG)	12.5	70.3/80.1	Mon.	0.16	n.d.	0.042	0.102

Fab: antigen-binding fragment; IgG: immunoglobulin G; n.d.: not determined; *T*_m_: melting temperature. *T*_m_1 and *T*_m_2 are the first and second apparent melting temperatures determined by differential scanning fluorimetry (DSF), respectively; *EC*_50_: half maximal effective concentration; *K*_D_: equilibrium dissociation constant; *IC_50_*: half maximal inhibitory concentration; Mon.: monomer; Agg.: aggregate; *IC*_50_^P^ and *IC*_50_^A^: *IC*_50_ determined by a pseudo-typed virus (D614 spike) and authentic SARS-CoV-2 virus, respectively.

## Data Availability

Data sharing is not applicable due to patent-pending and tech-transfer issue.

## Supplementary Materials

Supplementary materials can be found at https://www.mdpi.com/1422-0067/22/4/1913/s1.

## Abbreviations

mAb	Monoclonal antibody
SARS-CoV	Severe acute respiratory syndrome coronavirus
SARS-CoV-2	Severe acute respiratory syndrome coronavirus 2
MERS-CoV	Middle East respiratory syndrome coronavirus
ACE2	Angiotensin-converting enzyme 2
RBD	Receptor-binding domain
RBM	Receptor-binding motif
SARS-2 RBD	Receptor binding domain of severe acute respiratory syndrome coronavirus 2
ELISA	Enzyme-linked immunosorbent assay
HRP	Horseradish peroxidase
*EC*_50_	Half maximal effective concentration
IgG	Immunoglobulin G
Fab	Antigen-binding fragment
SEC	Size-exclusion chromatography
PTS	Protein thermal shift
*T*_m_	Melting temperature
*K*_D_	Equilibrium dissociation constant
K_on_	Association constant
*K*_off_	Dissociation constant
IPTG	Isopropyl β-D-1-thiogalactopyranoside
PBS	Phosphate-buffered saline
SDS-PAGE	Sodium dodecyl sulfate-polyacrylamide gel electrophoresis
CDR	Complementarity-determining region
FR	Framework
VH	Heavy-chain variable domain
VL	Light-chain variable domain
CL	Light-chain constant domain
BLI	Bio-layer Interferometry
*IC*_50_	Half maximal inhibitory concentration
TCID_50_	Median tissue culture infectious dose
RLU	Relative luminescence unit
MFI	Mean fluorescence intensity
