# Supplementary material for: Neutralizing Human Antibodies against Severe Acute Respiratory Syndrome Coronavirus 2 Isolated from a Human Synthetic Fab Phage Display Library
Source: Int J Mol Sci. 2021 Feb 15;22(4):1913. doi: 10.3390/ijms22041913 (PMC7918989; doi:10.3390/ijms22041913)
Supplement: Supplementary file 1 [file ijms-22-01913-s001.pdf]

Supplementary Figure Legends

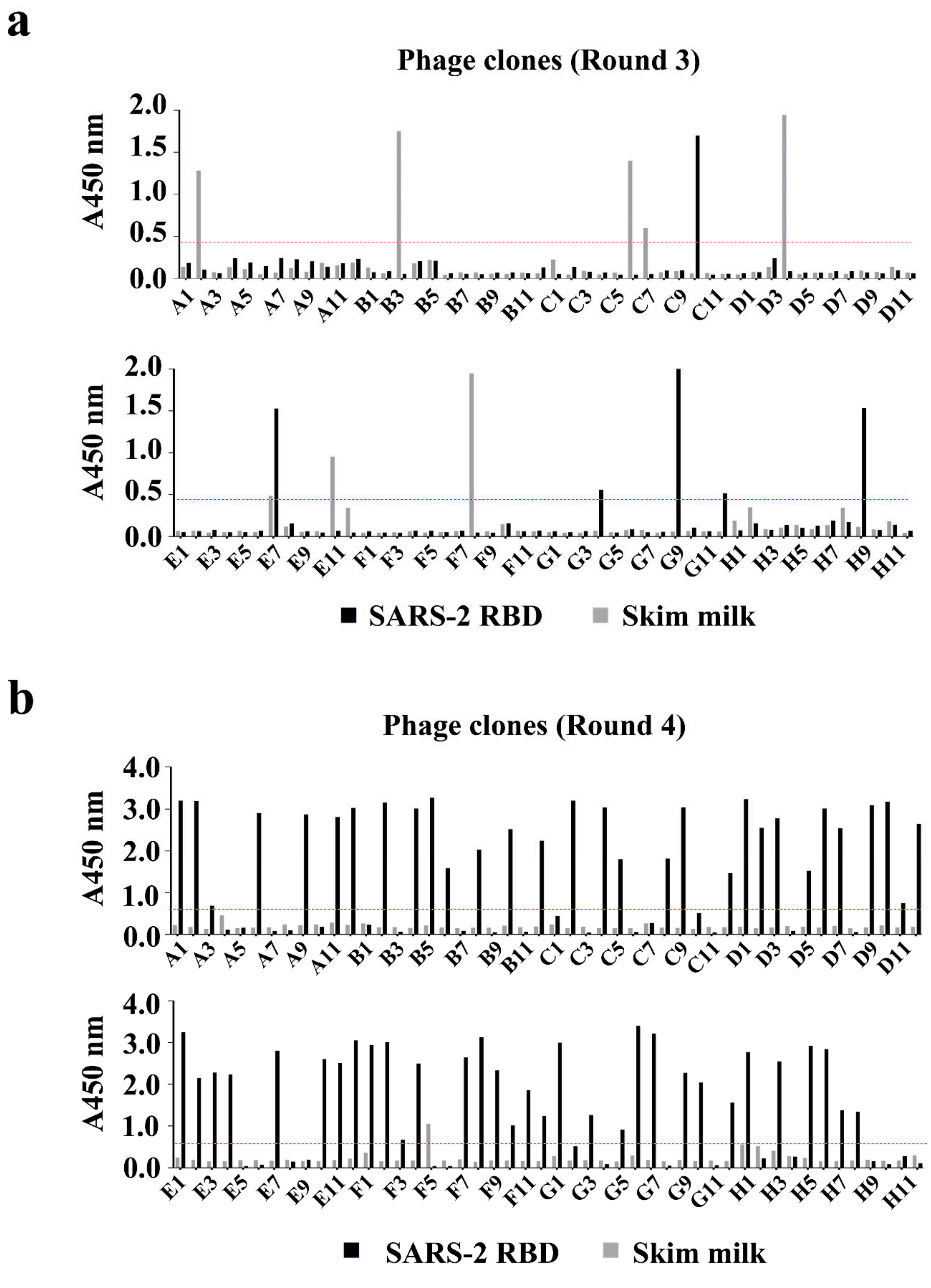

Figure S1. Phage ELISA against SARS-2 RBD with phages from each of the 3rd round (a) and 4th round (b) of the panning using the KFab-I library. The red dot lines indicate the cut off for selecting positive clones. Abbreviations: ELISA, enzyme-linked immunosorbent assay; A450 nm, Absorbance at 450 nm.

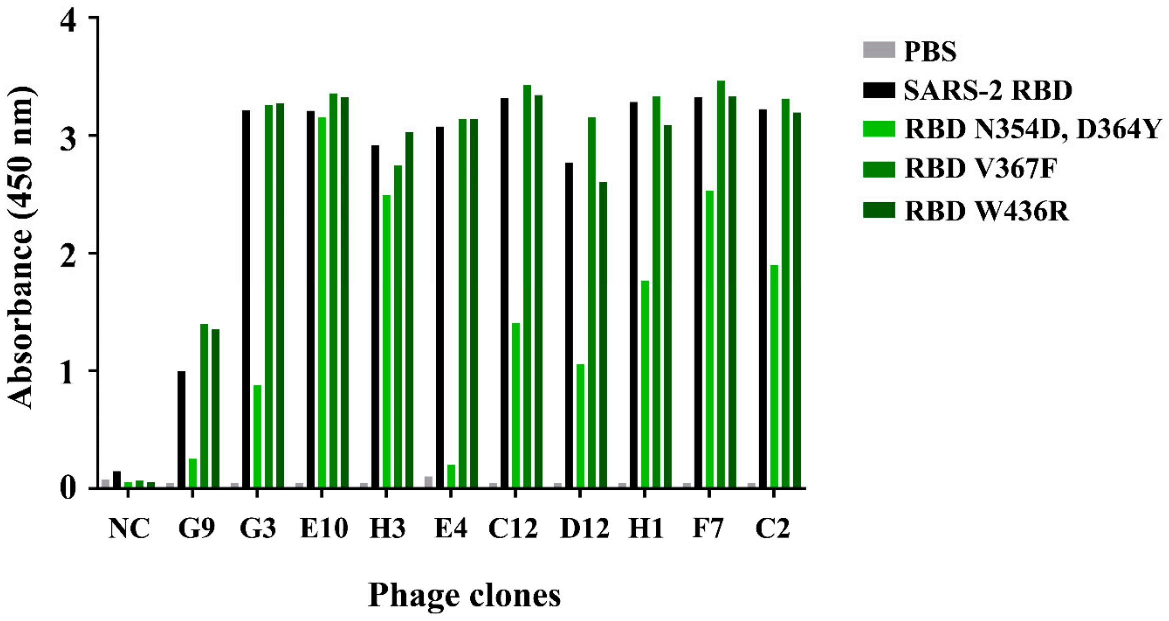

Figure S2. Monoclonal ELISA of 10 Fab phage clones against SARS-2 RBD and its variants. The numbers in each variants indicate positions on the RBD that mutations occurred. Abbreviations: N, Asparagine; D, Aspartate; W, Tryptophan; Y, Tyrosine; V, Valine; R, Arginine; F, Phenylalanine.

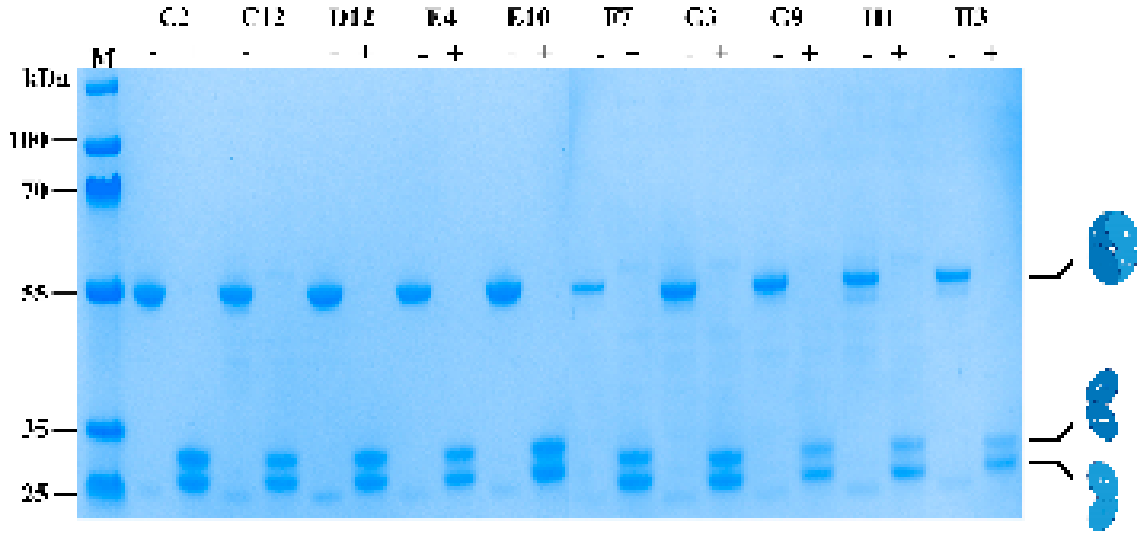

Figure S3. SDS-PAGE analysis of 10 human anti-SARS-2 RBD Fabs purified from periplasmic extracts of *E. coli*. + and – indicate with and without the reducing reagent (β-mercaptoethanol), respectively. kDa, kilodalton. The bars under kDa mark the positions of the molecular mass markers.

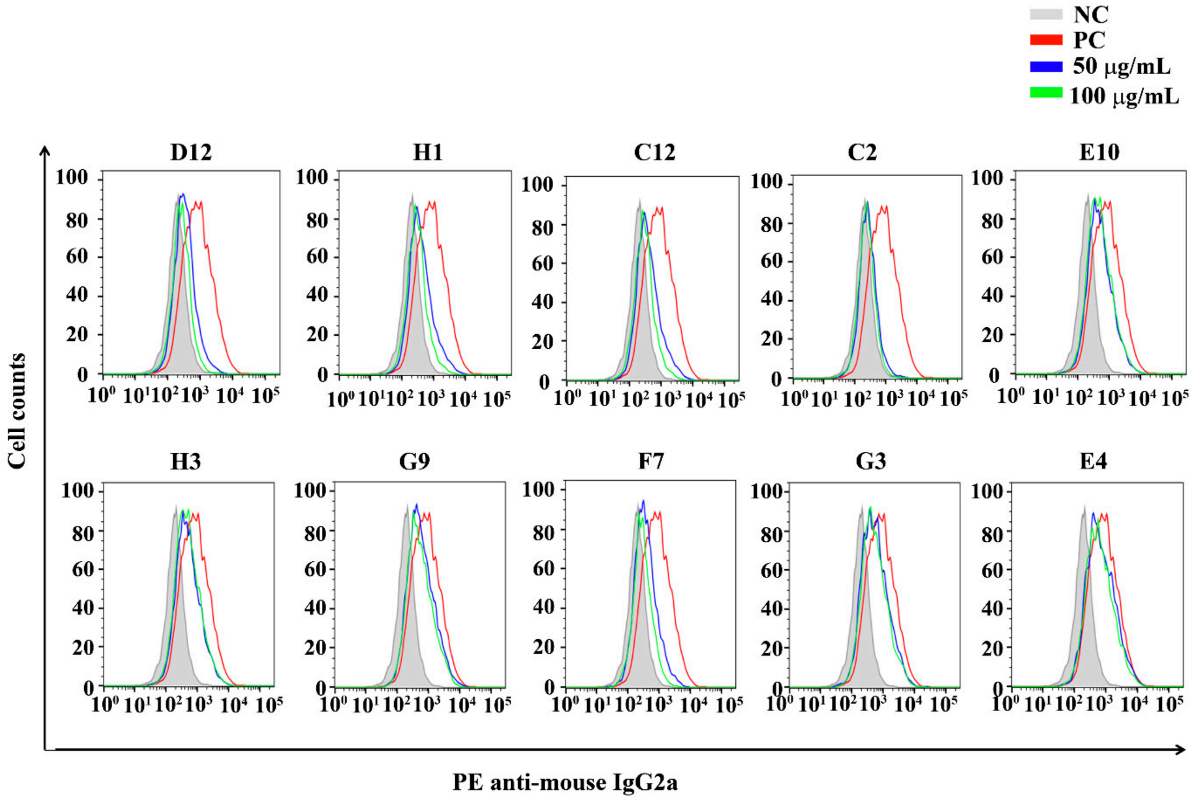

Figure S4. Flow cytometry analysis of blocking effect of human anti-SARS-2 RBD Fabs between SARS-CoV-2 RBD-mFc and ACE2-overexpressed cells. NC, a negative control, is cells only (grey line); PC, a positive control, is cells treated with SARS-CoV-2 RBD-mFc (red line); Blue and green lines indicate cells treated with mixture of SARS-CoV-2 RBD-mFc and anti-SARS-2 RBD Fabs of 50 μg/mL and 100 μg/mL, respectively.

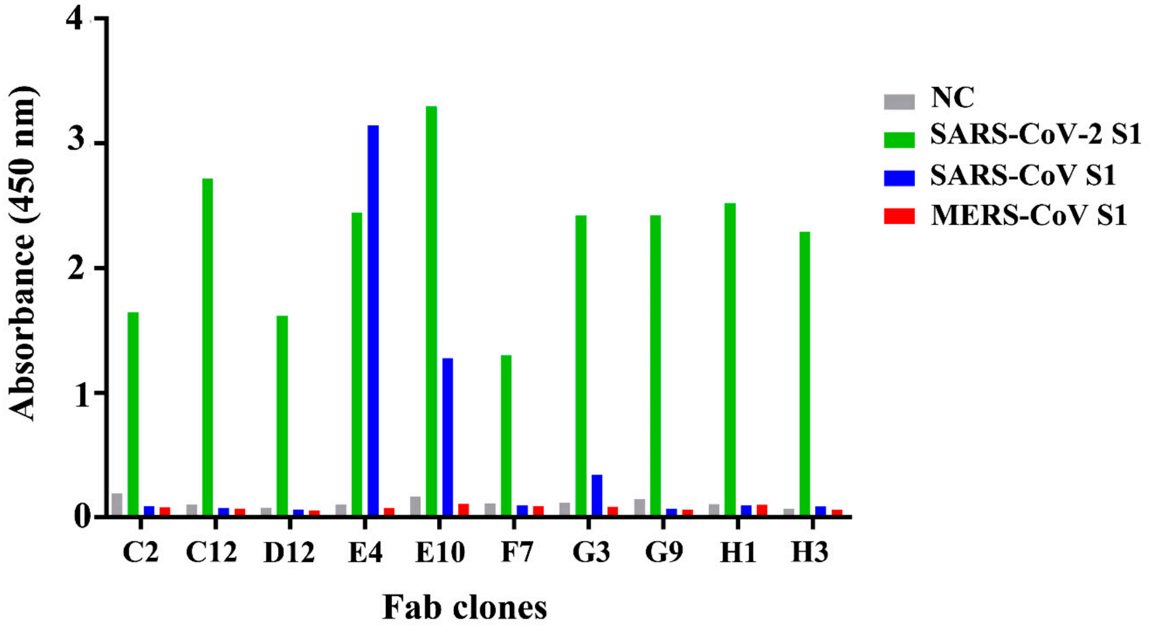

Figure S5. ELISA of 10 human anti-SARS-2 RBD Fabs against S1 proteins from SARS-CoV-2 (green), SARS-CoV (blue), and MERS-CoV (red).

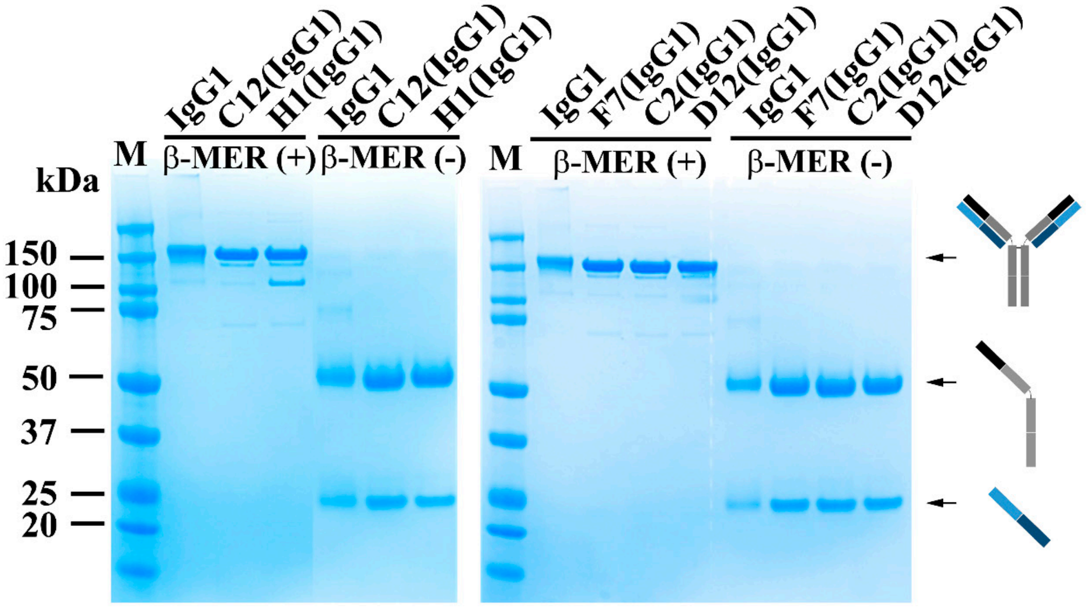

Figure S6. SDS-PAGE analysis of five human anti-SARS-2 RBD IgGs produced in HEK293 cells. β-MER (+) and β-MER (−) indicate with and without the reducing reagent (β-mercaptoethanol), respectively. kDa, kilodalton. The bars under kDa mark the positions of the molecular mass markers.

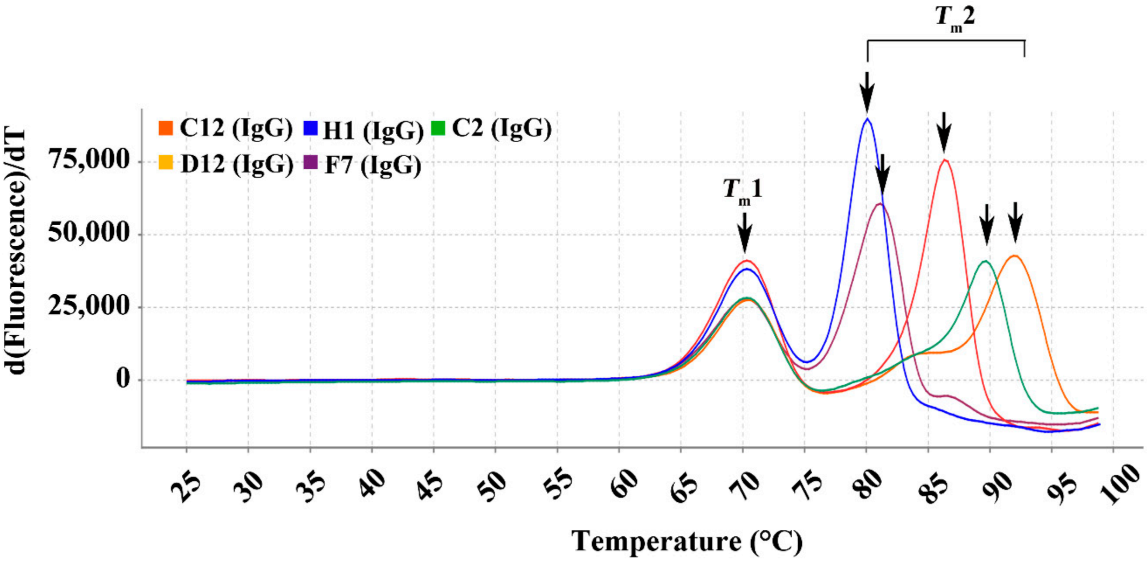

Figure S7. Determination of melting temperatures (T_ms) of five human anti-SARS-2 RBD IgGs using a PTS assay. T_m1 and T_m2 are the first and second apparent melting temperatures determined by a differential scanning fluorimetry (DSF), respectively.

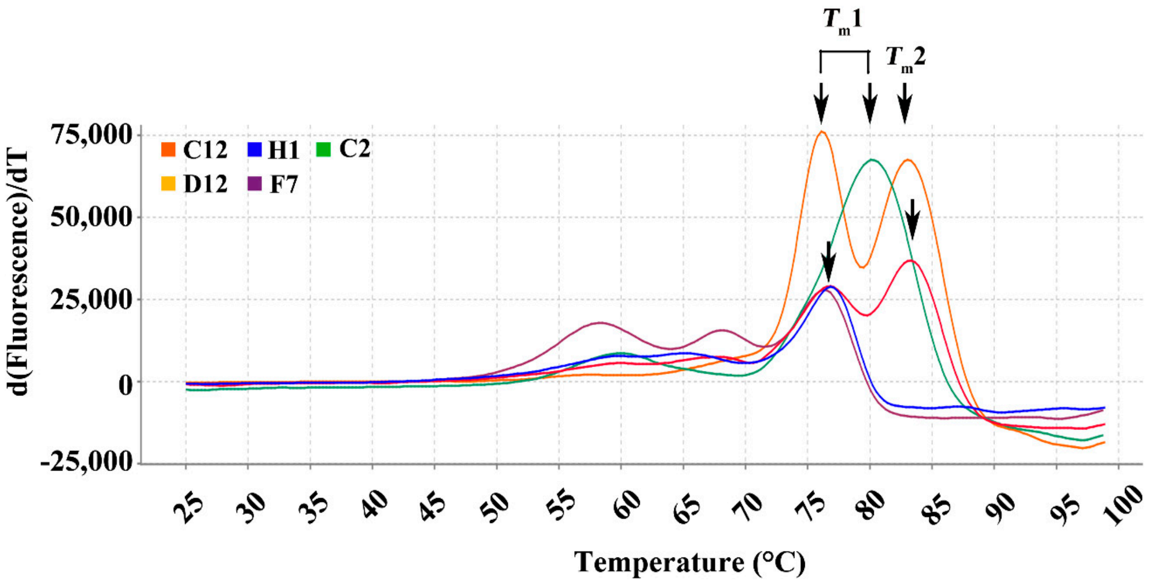

Figure S8. Determination of the melting temperatures (T_ms) of five human anti-SARS-2 RBD Fabs using a PTS assay. T_m1 and T_m2 are the first and second apparent melting temperatures determined by a differential scanning fluorimetry (DSF), respectively.

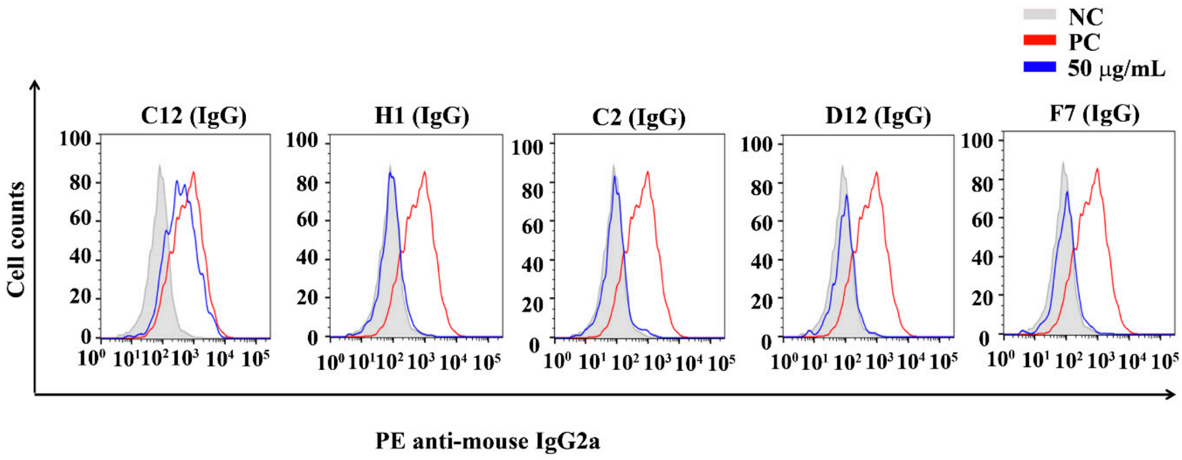

Figure S9. Flow cytometry analysis of blocking effect of human anti-SARS-2 RBD IgGs between SARS-CoV-2 RBD-mFc and ACE2-overexpressed cells. NC, a negative control, is cells only (grey line); PC, a positive control, is cells treated with SARS-CoV-2 RBD-mFc (red line); Blue line indicates cells treated with mixture of SARS-CoV-2 RBD-mFc and anti-SARS-2 RBD IgGs of 50 μg/mL.

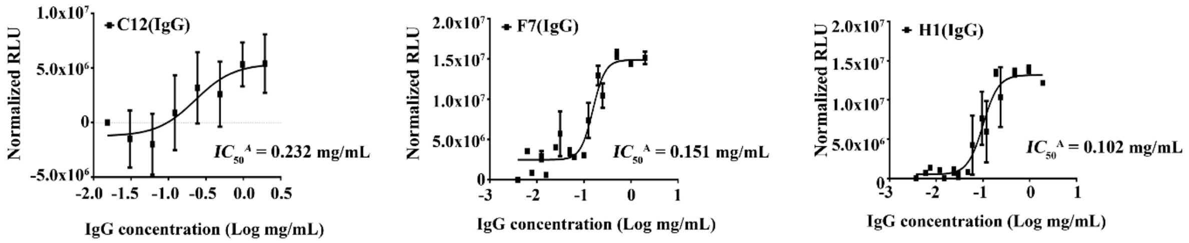

Figure S10. Neutralization assay of three human anti-SARS-2 RBD IgGs (C12, F7, and H1) on authentic SARS-CoV-2. Data presented as mean ± SE (SEM). Abbreviation: RLU, Relative luminescence units; IC_{50}^{A}, IC_{50} determined by authentic SARS-CoV-2 virus. RLU was normalized by subtracting values from the blank controls corresponding to each data points.

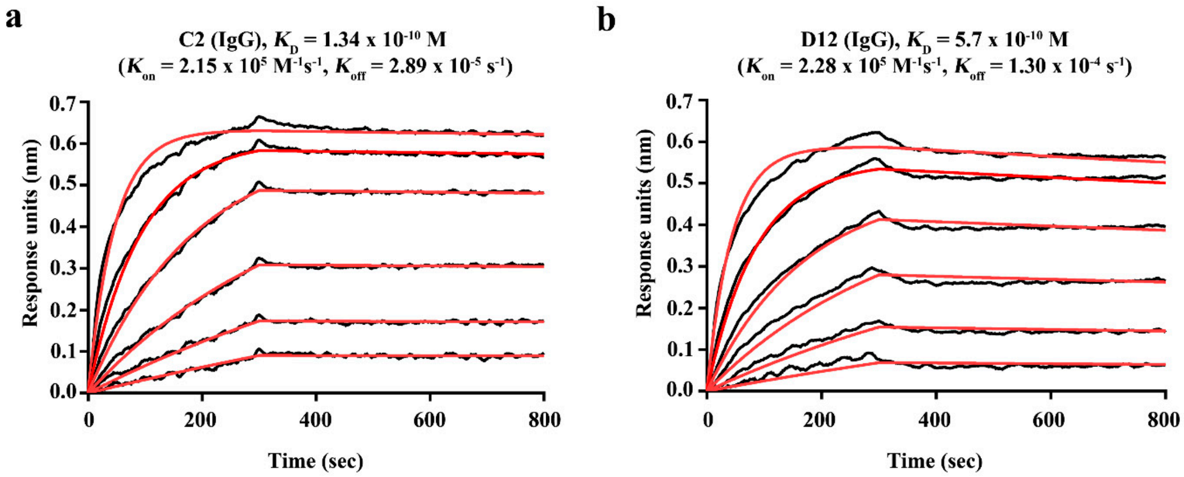

Figure S11. Affinity determination of human anti-SARS-2 RBD IgGs using a BLI (Octet). Black and red lines indicate data points measured from different concentrations and corresponding fitted curves, respectively. Abbreviation: K_{on} and K_{off}, association and dissociation constants, respectively; K_D, equilibrium dissociation constant.
